# Supplementary material for: Hand position fields of neurons in the premotor cortex of macaques during natural reaching
Source: Nat Commun. 2025 Apr 12;16:3489. doi: 10.1038/s41467-025-58786-3 (PMC11993741; doi:10.1038/s41467-025-58786-3)
Supplement: Supplementary file 1 — Supplementary Information [file 41467_2025_58786_MOESM1_ESM.pdf]

Supplementary Information

Hand Position Fields of Neurons in the Premotor Cortex of Macaques during Natural Reaching

Spatial organization of positional preferences

To investigate the spatial organization of hand position-tuned cells on the arrays, we first set the max firing point in the spatial firing rate map as neuron's hand position preference. Then we labeled the detected hand position preferences for each electrode in the array. To make the pattern more evident, we combined the array results from all four monkeys (Supplementary Fig. 9).

At each electrode site, if a hand position-tuned cell was recorded, we used colored markers to indicate its positional preference relative to the monkey's body (set as x=0, y=0, color white). Red denotes the left-side position of the monkey and green denotes the right-side position of the monkey. Saturation increases from the midline towards the left/right reaching boundaries, and brightness decreases from near area to far area. We analyzed the position preferences of all 132-hand position-tuned cells. When merging results from different monkeys, we used the arcuate sulcus (AS) as the reference for alignment.

As illustrated in the figure, red markers dominate at the more dorsal electrodes, while green markers are predominant at the more ventral electrodes. This indicates that, along the dorsal-ventral axis, the preferred hand positions of neurons shift from the left side of the body to the right side. Furthermore, markers near the AS have higher saturation and lower brightness, indicating the position preferences at the reaching limits. Markers away from the AS have lower saturation and higher brightness, indicating that the position preferences locate close to the midline.

Stability of hand position coding across states

Because the reaching movements were towards moving targets, it would be intriguing to assess the effect of the dynamic movement planning, such as inhibitory control[1], on the hand position coding in premotor cortex. We chose to explore the stability of coding under different states of food/target.

The moving state of food was not constant during single trial, so there were no perfect static-target trials or perfect moving-target trials. Instead, we define the time periods when the food's moving speed was lower than 1 cm/s as the target static periods and the remaining time periods as the target moving periods. The target static periods accounted for only 2.2 ± 1.6% of each recording session, making it difficult to calculate a valid hand position spatial firing rate map, as in the main text. As a result, we could not determine the stability of hand position coding by directly comparing spatial firing rate maps from two periods. Instead, we decided to utilize a trajectory decoding test:

First, we divided the target moving periods into the training set (70%) and the testing set (30%). Second, we trained a hand trajectory decoder (Kalman Filter, details in Methods) with the training set data and calculated the decoding performance in the test set. Third, we directly applied the same trained decoder to the target static periods to assess the performance. To get the random performance, we first randomly shifted the spike trains in time to disrupt the correspondence of neural activity with the target moving states, then we performed the same decoding procedure as above to calculate one decoding accuracy. We repeated 100 times for each session and the average accuracy was calculated as a random result for each session.

The results showed that decoders extracted from the target moving periods performed even greater in the target static periods (Supplementary Fig. 10A), which revealed the stability of the hand position coding.

Similarly, we divided each session into hand moving periods and hand static periods and then performed the same analysis process and arrived at a similar conclusion (Supplementary Fig. 10B).

These two results together proved that populational hand position coding during the reach-and-grasp task was stable and was not affected by the food/target state or the hand state.

Stability of hand position coding across time

Specific to the Utah array, we adopted Fraser et al.'s method[2], which integrated pairwise cross-correlogram, autocorrelogram, waveform shape, and mean firing rate as identifying features of a neuron. When comparing two units recorded on different sessions using these features, their similarity scores typically fell into one of two categories: high, suggesting that the recordings came from the same unit, or low, indicating that they originated from different units.

Out of the 839 putative single units extracted in our experiments, this method identified 451 (Monkey A-30, B-54, X-238, Z-129) unique cells. Further, 53.4% (241/451) of these unique cells were recorded in only one session and 46.6% (210/451) were recorded repeatedly over multiple sessions.

Out of the 451 unique cells, we selected 340 cells with stable firing rate maps and identified 98 hand position tuned cells following the same process in the main text. Based on this result, the proportion of hand-position tuned cells in the PMd came rise to 28.5%. Further, the proportion of primary hand-position tuned cells came to 11.8%.

We next explored whether the properties of these hand position tuned cells would change over time. To quantify the stability of the hand position tuned cells, we calculated the correlation between maps of the same cells recorded in different sessions. Also, we calculated the distance

between the peak value position of the maps of the same cell recorded in different sessions to measure the drift of this cell's position preference over time. For comparison, we randomly sampled pairs of different cells and then computed the above two metrics and sampled 100 such pairs for each monkey. Before the calculations, maps from different sessions were aligned according to the reference points (i.e. monkeys' body positions).

Comparisons showed that the identified unique cells had significantly stable hand spatial firing rate maps across sessions ($P = 1.32 \times 10^{-14}$, $n = 41$ cell-pairs for same, $n = 400$ cell-pairs for different, Kruskal-Wallis test) and significantly smaller drift in position preferences ($P = 2.02 \times 10^{-5}$, $n = 41$ cell-pairs for same, $n = 400$ cell-pairs for different, Kruskal-Wallis test, Supplementary Fig. 11). It is important to note that we did not use hand spatial firing rate maps and peak value positions as features when tracking unique cells across sessions. The results indicated the stability of hand position-tuned cells across sessions/time.

Searching for grid-like cells with periodic hexagonal spaced fields in PMd

To assess whether the hexagonal firing patterns characteristic of grid cells were present in the PMd cortex, we calculated grid scores based on rotated autocorrelograms (see Grid Score below). Only one cell had a grid score above the 99th percentile of shuffled grid scores (within the horizontal plane), and this percentage was not significantly higher than the chance level (Supplementary Fig. 12; $p = 0.98$, Binomial test with expected $P_0 = 0.01$). Analyses of the other two planes give the same conclusion. Future investigations should encompass a broader range of recording sites within the motor control circuit to explore whether such cell type exists.

Grid Score. First, we calculated a spatial autocorrelogram based on Pearson's product-moment correlation coefficient with corrections for edge effects and unvisited locations. In practice, the autocorrelogram was estimated as:

$$\begin{aligned} S_1 &= \sum \lambda(x, y)\lambda(x - \tau_x, y - \tau_y) \\ S_2 &= \sum \lambda(x, y) \\ S_3 &= \sum \lambda(x - \tau_x, y - \tau_y) \\ S_4 &= \sum \lambda(x, y)^2 \\ S_5 &= \sum \lambda(x - \tau_x, y - \tau_y)^2 \\ r(\tau_x, \tau_y) &= \frac{nS_1 - S_2 S_3}{\sqrt{nS_4 - S_2^2}\sqrt{nS_5 - S_3^2}} \end{aligned} \tag{1}$$

where $\lambda(x, y)$ is the average firing rate at position (x, y), τ_x and τ_y are spatial lags and n is the number of pixels in $\lambda(x, y)$ for which firing rate estimated for both $\lambda(x, y)$ and $\lambda(x - \tau_x, y - \tau_y)$. Autocorrelations were not calculated for lags (τ_x, τ_y) where $n < 20$.

The gird score was based on calculating the Pearson correlations between the autocorrelogram and its rotated version [3], which was a modified version of methods used in [4–7]. First, a series of rings centered on the central field in the autocorrelogram were determined, which has the same inner radius r_i and various out radius r_o. r_i was defined as either the first local minimum in a curve showing correlation as a function of the average distance from the center or as the first incidence where the correlation was negative, whichever happened first. r_o was increasing from a minimum of 4 bins more than the inner radius to a maximum of 4 bins less than half of the behavioral border length. The Pearson correlations between the original ring and its rotated versions were calculated. In one group, the rings were rotated for 60 and 120 degrees, and in the other group, the rings were rotated for 30, 90, and 150 degrees. For each ring, the minimum difference, τ, between any of the correlation coefficients in the first group and that in the second group was saved. Then the grid score was defined as the largest τ among all rings.

Supplementary Table 1. Detailed experimental information. Subjects, dates, task durations, frame rates for motion tracking, and the number of putative single units of each experiment session.

Subject	Date	Duration	Frame rate	Sorted units
Monkey A	2022.01.12	25m8s	60	16
	2022.01.13	27m46s		16
	2022.01.14	21m34s		19
Monkey B	2021.07.07	34m17s	100	37
	2021.07.08	35m42s		22
	2021.07.09	31m49s		42
Monkey X	2021.06.23	22m21s	75	81
	2021.07.01	9m27s	100	89
	2021.07.07	34m20s		90
	2021.07.08	29m33s		130
Monkey Z	2021.10.16	21m6s	100	51
	2021.10.17	28m37s		53
	2021.10.18	19m47s		51
	2021.10.19	21m21s		23
	2021.12.29	28m12s	60	35
	2021.12.30	28m11s		31
	2021.12.31	31m10s		29
	2022.01.05	22m32s		24

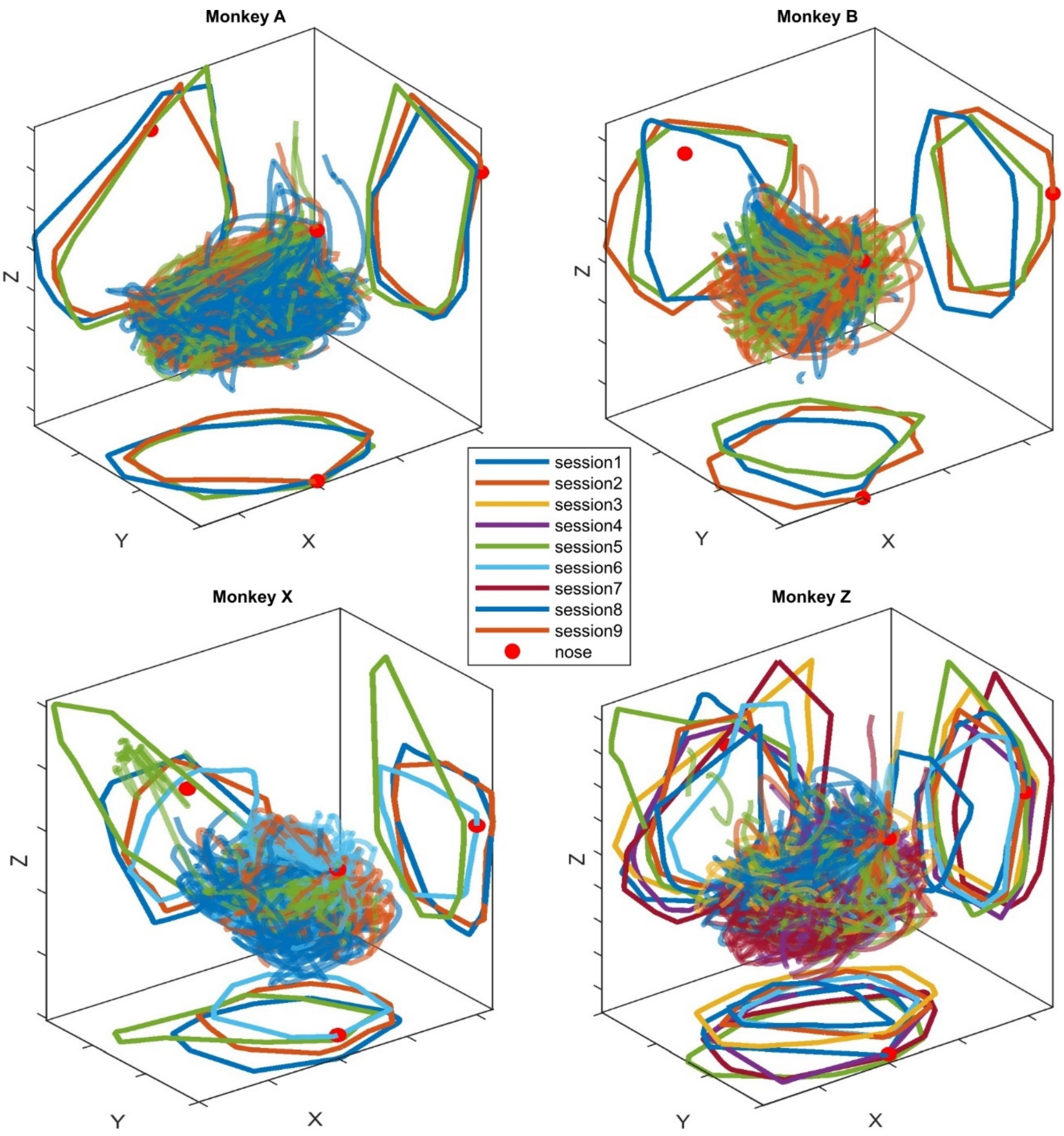

Supplementary Fig. 1. The spatial trajectories of the food/targets. We plot the trajectories of the food with reference to the position of monkey's nose from all sessions into the same panel. Different colors indicate different sessions. The red dot indicates the monkey's nose. Both trajectories in 3D space and boundaries projected into three 2D planes are shown. The experimenter made sure that targets were within the reach of monkeys most of the time. Source data are provided as a Source Data file.

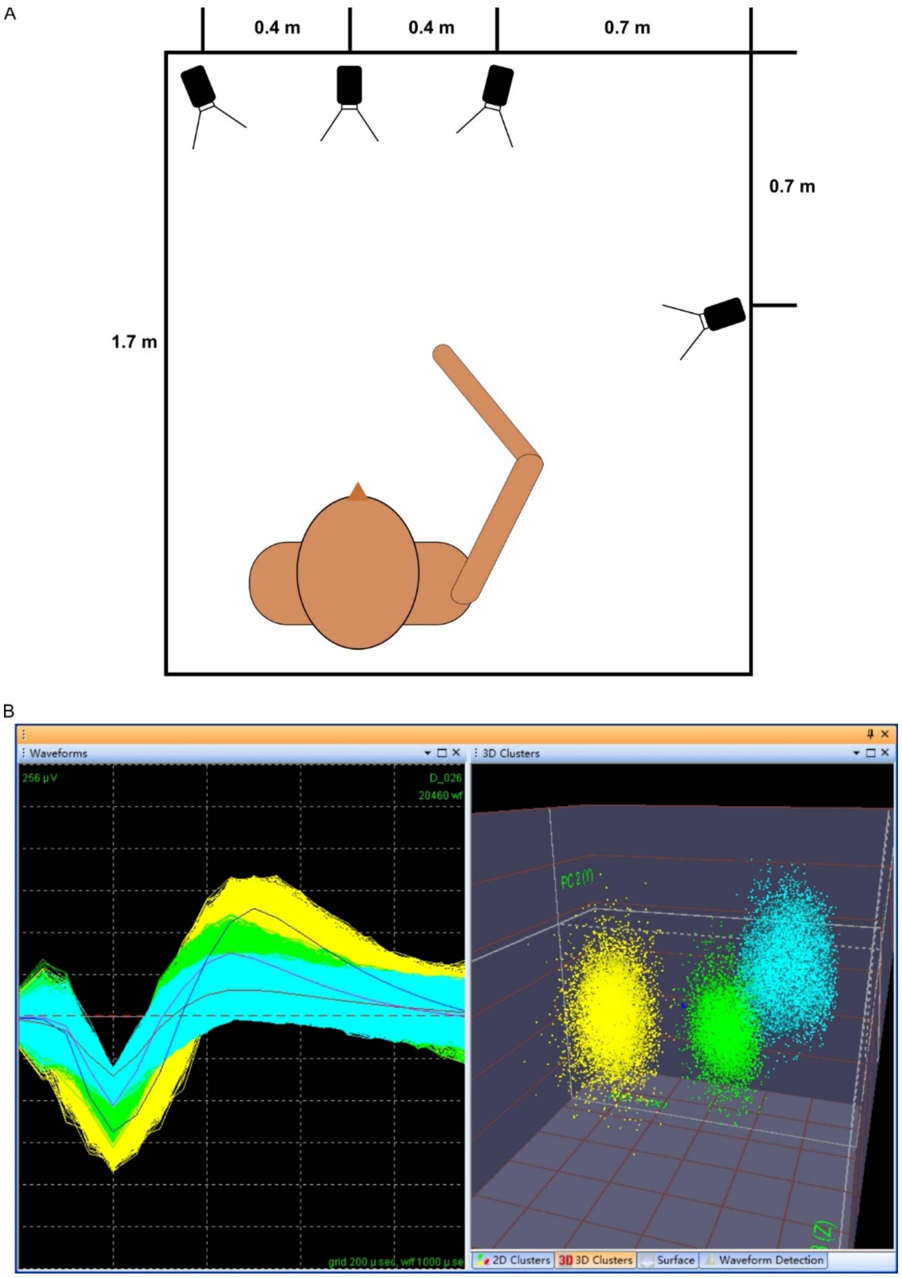

Supplementary Fig. 2. Camera setting ups and spike sorting criteria. (**A**) The top view of the motion tracking setup, cameras are ~1 m above the ground. (**B**) Spike sorting illustration from one example electrode, waveforms and first 3 PC clusters of the three well-isolated units in this electrode are shown.

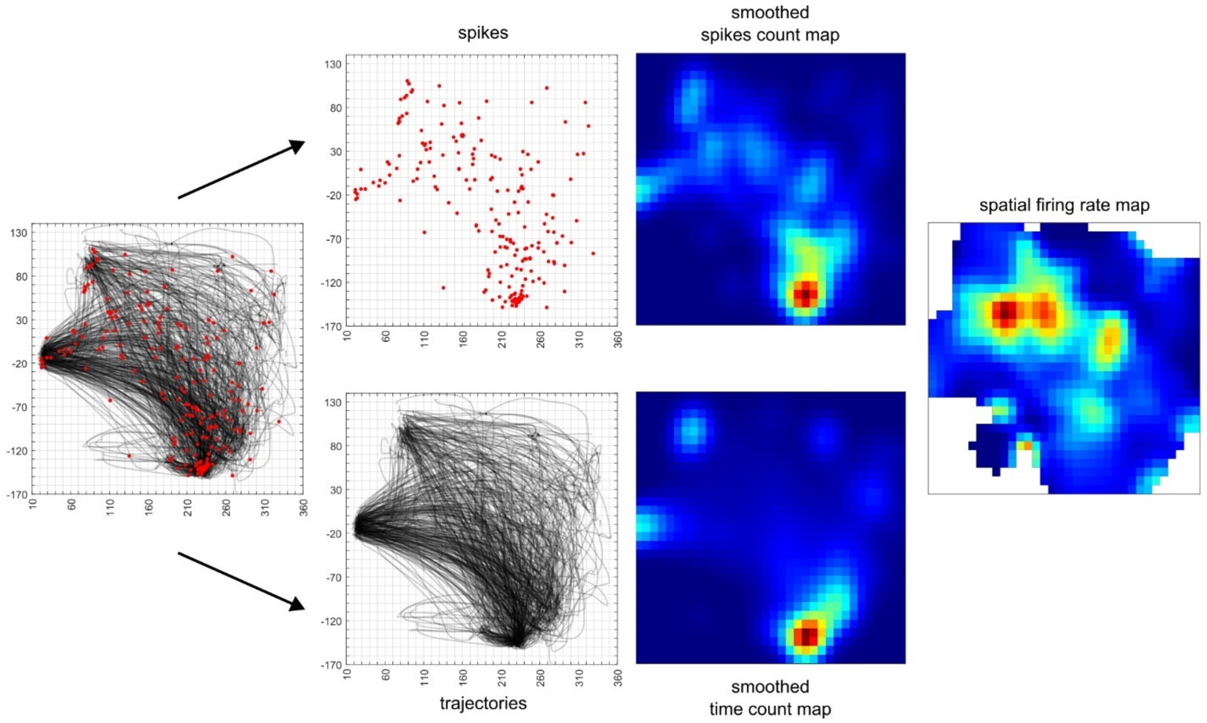

Supplementary Fig. 3. Illustration of the intermediate steps of the calculating spatial firing rate maps. Details are described in Methods. We first divided the moving space into grided spatial bins (bin size: 1cm × 1cm, column 1 and column 2). Next, we counted the number of spikes and the time in each bin respectively and smoothed the spike count map and the time map using a Gaussian filter (std: 1.5, column 3). Finally, we divided the smoothed spike count map by the smoothed time map to get the spatial firing rate map (column 4). Source data are provided as a Source Data file.

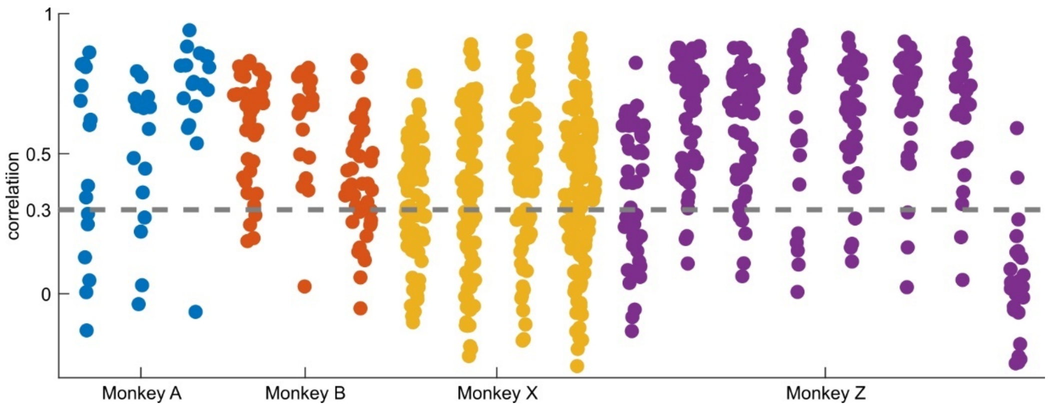

Supplementary Fig. 4. Distribution of cells' spatial firing rate map stability in each session. We set 0.3 (dashed line) as the stability threshold. Most sessions of Monkey A (blue dots), B (red dots), and Z (purple dots) showed a bimodal distribution, with 0.3 being a suitable threshold to remove the small peak. The distributions of Monkey X (yellow dots) were unimodal, and we utilized the same threshold to remove neurons with bad recording stabilities only. Source data are provided as a Source Data file.

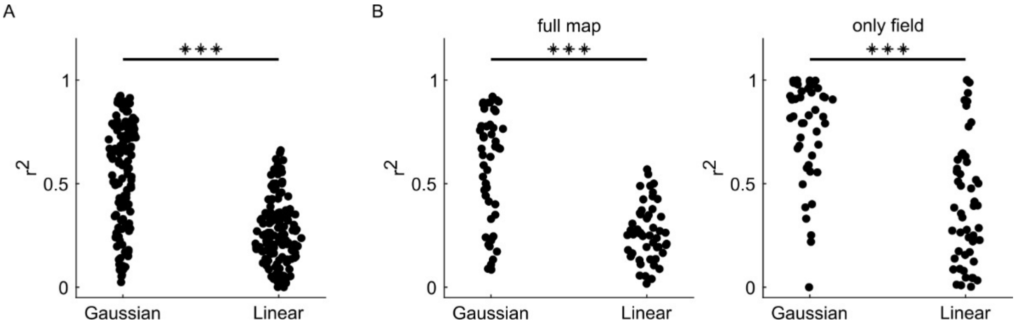

Supplementary Fig. 5. Comparisons between gaussian fittings and linear fittings. (**A**) Results in full map data still confirmed the superiority of the Gaussian fitting ($P = 3.02 \times 10^{-26}$, $n = 132$ cells, one-sided paired t-test). (**B**) Comparison of fitting results in the primary hand position-tuned cells. The firing rate of primary hand position-tuned cells ($n = 50$) was better described as a Gaussian function of hand position rather than a liner plane. Left, fitting in full map data ($P = 9.35 \times 10^{-12}$). Right, fitting position field data ($P = 2.49 \times 10^{-11}$). *** $P < 0.001$, one-sided paired t-test. Source data are provided as a Source Data file.

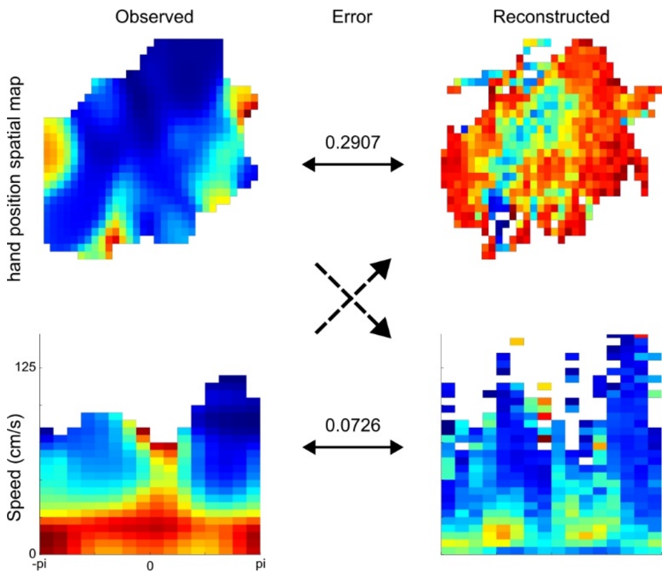

Supplementary Fig. 6. Illustration of the reconstruction analysis. Details are described in Methods. Upper row: spatial firing rate maps. Bottom row: speed-direction joint firing rate maps. Left column: observed firing rate maps. Right column: reconstructed firing rate maps. We first assumed that the cell is a pure hand position-tuned cell and based on this assumption and monkey's actual hand moving parameters, we reconstructed what would be the expected speed-direction tuning (upper left to bottom right); and conversely, we assumed that the cell is a pure speed-direction cell and reconstructed the expected hand position tuning (bottom left to upper right). The dashed arrows indicate the direction of reconstruction. Normalized mean squared errors were computed between the observed and reconstructed firing rate maps and indicated between the maps, and the final hand-position/speed-direction index of this cell is 4.0. The calculation of hand-position/food-location index followed a similar process by setting the food-location tuning as the opposing tuning property. Source data are provided as a Source Data file.

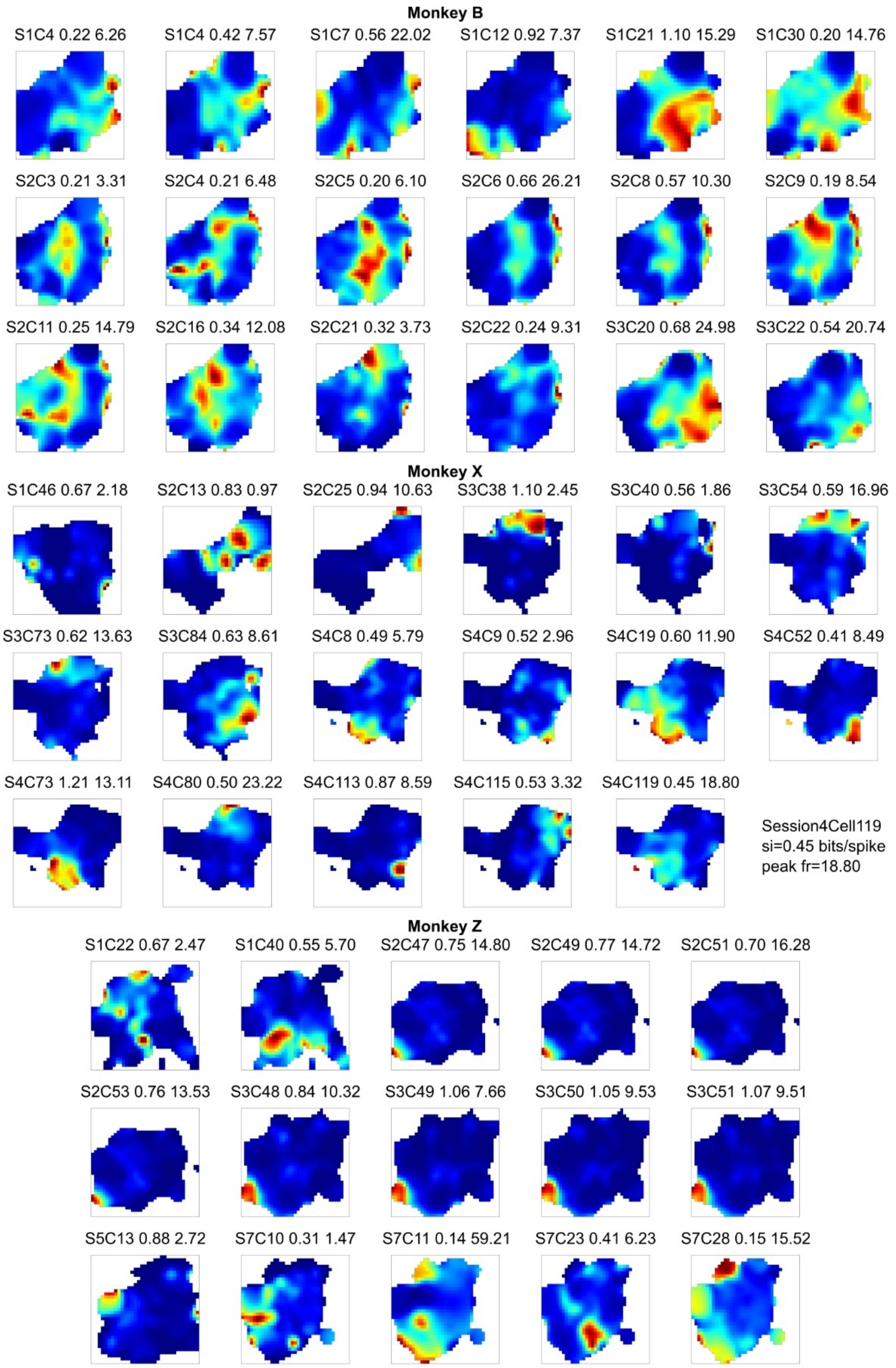

Supplementary Fig. 7. Spatial firing rate maps for primary hand position-tuned cells (*n* = 50). Dark blue indicates the minimal firing rate within each map and dark red indicates the

maximal firing rate within each map. Labels, spatial information, and peak firing rate of each cell are marked on the top of maps. The location of the monkey’s nose (not actually shown in the figure) is around the left middle of each map, facing right. There are no primary hand position-tuned cells in monkey A. Source data are provided as a Source Data file.

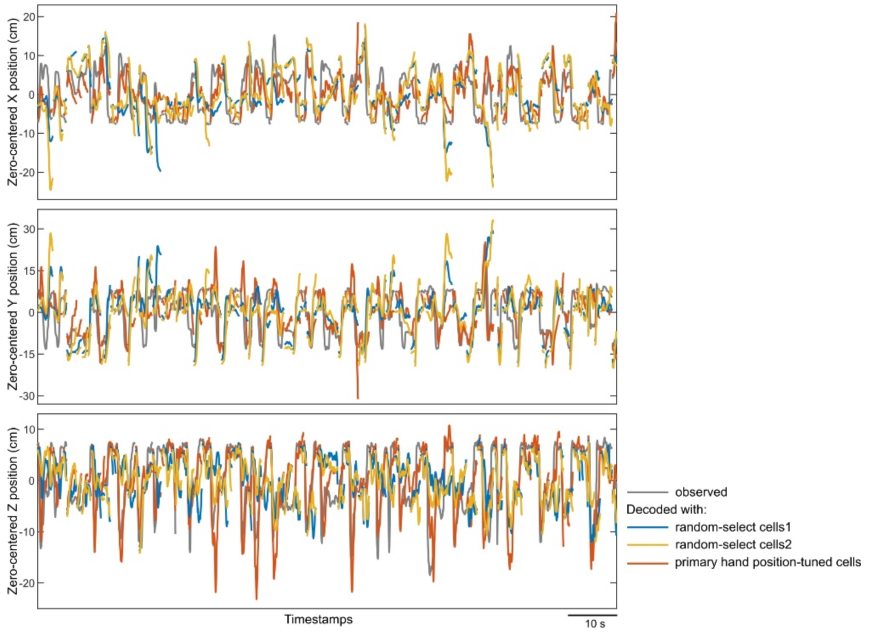

Supplementary Fig. 8. Examples of observed and decoded hand trajectories using random-select cells and primary hand position-tuned cells. Gray lines are the observed trajectories, blue lines are the decoded trajectories using random-select cells1, yellow lines are the decoded trajectories using random-select cells2, and red lines are the decoded trajectories using primary hand position-tuned cells. The results shown are based on the same dataset as Fig. 3G. The horizontal scale bar represents 10 s. Source data are provided as a Source Data file.

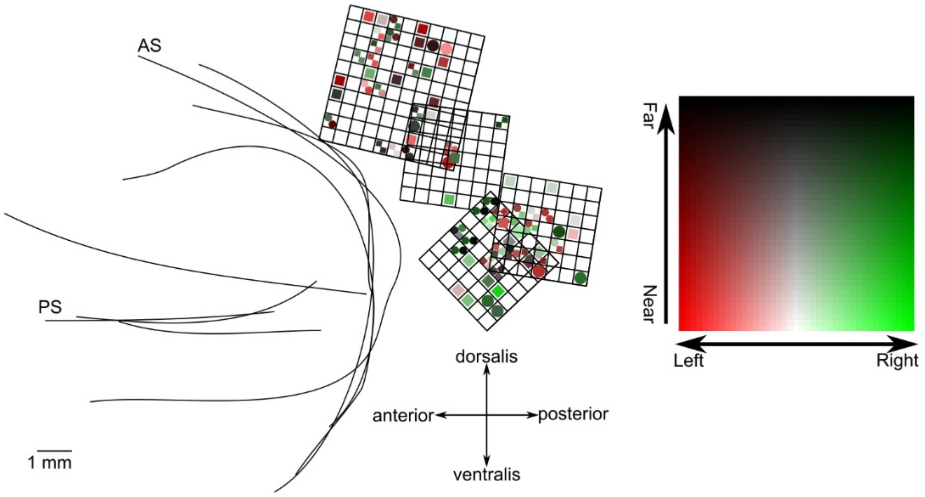

Supplementary Fig. 9. Spatial organization of hand position preferences summarized from all monkeys and all sessions. Left panel, circular markers represent hand position preferences of primary hand position-tuned cells (*n* = 50), and square markers represent hand position preferences of remaining hand position-tuned cells (*n* = 82). If multiple hand position-tuned cells were recorded on the same electrode site, the markers sizes are adjusted to ensure that these markers remain within a single grid. Right panel, the marker colors correspond to position preferences relative to the body. Red denotes the left side of the monkey and greed denotes the right side of the monkey. Saturation increases from the midline towards the left/right reaching boundaries, and brightness decreases from near area to far area. Monkey's body positions were set as color white. Source data are provided as a Source Data file.

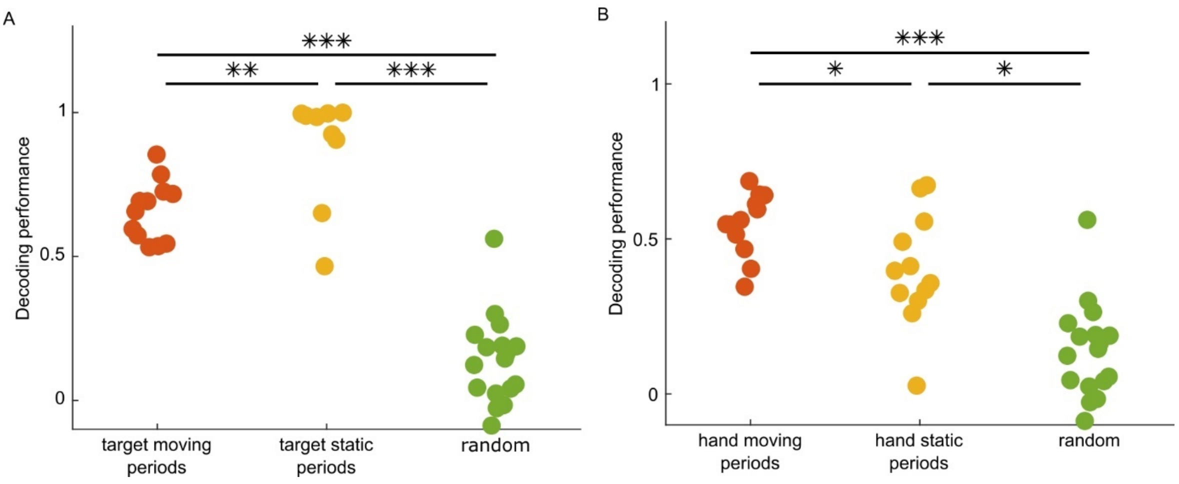

Supplementary Fig. 10. Comparison of decoding performance across states. (**A**) The decoding performances in the target static periods (yellow dots) were greater than those in target moving periods (red dots) using the same decoders trained in target moving periods ($P = 0.0076$) and greater than the random performances (green dots) ($P = 9.53 \times 10^{-8}$). The decoding performances in the target moving periods were greater than the random performances ($P = 3.30 \times 10^{-8}$). (**B**) The decoding performances in the hand static periods were lower than those in hand moving periods using the same decoders trained in hand moving periods ($P = 0.016$) but greater than the random performances ($P = 0.0119$). The decoding performances in the hand moving periods were greater than the random performances ($\mathrm{P} = 4.83 \times 10^{-6}$). * $P < 0.05$, ** $P < 0.01$, *** $P < 0.001$, $n = 18$ sessions, one-sided paired t-test. Source data are provided as a Source Data file.

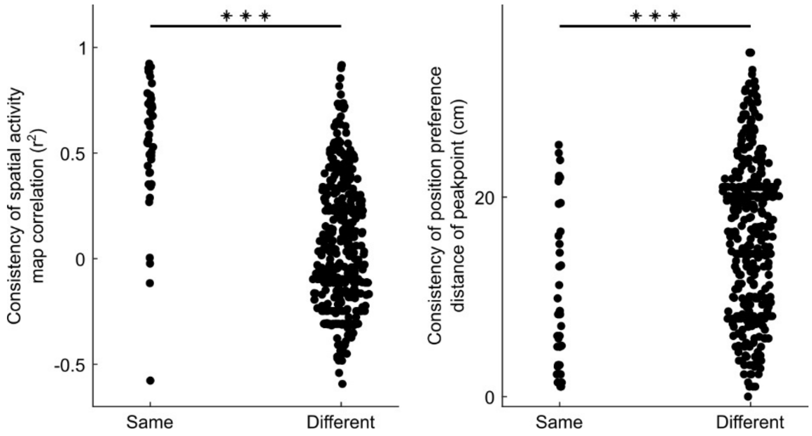

Supplementary Fig. 11. Comparisons of the consistencies of spatial activity (left panel) and position preferences (right panel) between the same (*n* = 41 pairs) or different units (*n* = 400 pairs) recorded in different sessions. If a cell was identified being repeatedly recorded across sessions, it had significantly stable spatial firing rate maps ($P = 1.32 \times 10^{-14}$) and significantly smaller drift in its preferred hand position ($P = 2.02 \times 10^{-5}$). *** $P < 0.001$, Kruskal-Wallis test. Source data are provided as a Source Data file.

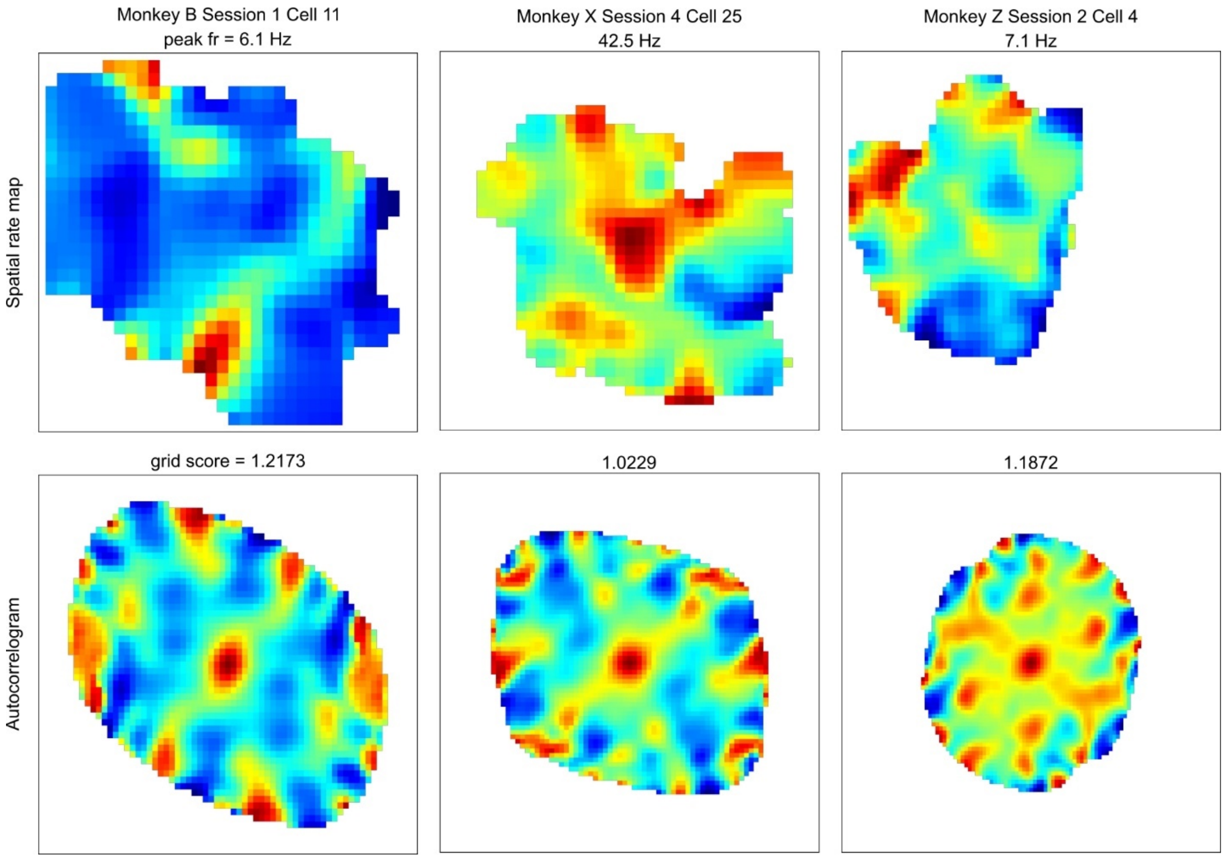

Supplementary Fig. 12. There were few grid-like cells in PMd. 1 grid cell in the XY plane (from Monkey B) and 2 grid cells in the XZ plane (from Monkey X and Z) were identified. The top panels show the 2D firing rate maps with the peak firing rates indicated. The bottom panels show corresponding autocorrelations with the grid scores. Source data are provided as a Source Data file.

Supplementary References

1. Pani, P. *et al.* Neuronal population dynamics during motor plan cancellation in nonhuman primates. *Proceedings of the National Academy of Sciences* **119**, (2022).
2. Fraser, G. W. & Schwartz, A. B. Recording from the same neurons chronically in motor cortex. *J Neurophysiol* **107**, 1970–1978 (2012).
3. Langston, R. F. *et al.* Development of the Spatial Representation System in the Rat. *Science (1979)* **328**, 1576–1580 (2010).
4. Mao, D. *et al.* Spatial modulation of hippocampal activity in freely moving macaques. *Neuron* **109**, 3521-3534.e6 (2021).
5. Grieves, R. M. *et al.* The place-cell representation of volumetric space in rats. *Nat Commun* **11**, 789 (2020).
6. Long, X. & Zhang, S.-J. A novel somatosensory spatial navigation system outside the hippocampal formation. *Cell Res* **31**, 649–663 (2021).
7. Kropff, E., Carmichael, J. E., Moser, M.-B. & Moser, E. I. Speed cells in the medial entorhinal cortex. *Nature* **523**, 419–424 (2015).
